# Jasmonates and Ethylene Shape Floridoside Synthesis during Carposporogenesis in the Red Seaweed *Grateloupia imbricata*

**DOI:** 10.3390/md22030115

**Published:** 2024-02-28

**Authors:** Pilar Garcia-Jimenez, Diana del Rosario-Santana, Rafael R. Robaina

**Affiliations:** Department of Biology, Faculty of Marine Sciences, Instituto Universitario de Investigación en Estudios Ambientales y Recursos Naturales i-UNAT, Universidad de Las Palmas de Gran Canaria, 35017 Las Palmas, Spain; diana.delrosario@ulpgc.es (D.d.R.-S.); rafael.robaina@ulpgc.es (R.R.R.)

**Keywords:** ethylene, floridoside, galactose-1-phosphate uridyltransferase, α-galactosidase, jasmonates, red seaweed, sulfated galactans

## Abstract

Floridoside is a galactosyl–glycerol compound that acts to supply UDP-galactose and functions as an organic osmolyte in response to salinity in Rhodophyta. Significantly, the UDP-galactose pool is shared for sulfated cell wall galactan synthesis, and, in turn, affected by thallus development alongside carposporogenesis induced by volatile growth regulators, such as ethylene and methyl jasmonate, in the red seaweed *Grateloupia imbricata*. In this study, we monitored changes in the floridoside reservoir through gene expression controlling both the galactose pool and glyceride pool under different reproductive stages of *G. imbricata* and we considered changing salinity conditions. Floridoside synthesis was followed by expression analysis of *galactose-1-phosphate uridyltransferase* (*GALT*) as UDP-galactose is obtained from UDP-glucose and glucose-1P, and through *α-galactosidase* gene expression as degradation of floridoside occurs through the cleavage of galactosyl residues. Meanwhile, glycerol 3-phosphate is connected with the galactoglyceride biosynthetic pathway by glycerol 3-phosphate dehydrogenase (G3PD), monogalactosyl diacylglyceride synthase (MGDGS), and digalactosyl diacylglyceride synthase (DGDGS). The results of our study confirm that low *GALT* transcripts are correlated with thalli softness to locate reproductive structures, as well as constricting the synthesis of UDP-hexoses for galactan backbone synthesis in the presence of two volatile regulators and methionine. Meanwhile, *α-galactosidase* modulates expression according to cystocarp maturation, and we found high transcripts in late development stages, as occurred in the presence of methyljasmonate, compared to early stages in ethylene. Regarding the acylglyceride pool, the upregulation of *G3PD*, *MGDGS*, and *DGDGS* gene expression in *G. imbricata* treated with MEJA supports lipid remodeling, as high levels of transcripts for *MGDGS* and *DGDGS* provide membrane stability during late development stages of cystocarps. Similar behavior is assumed in three naturally collected thalli development stages—namely, fertile, fertilized, and fertile—under 65 psu salinity conditions. Low transcripts for *α-galactosidase* and high for *G3PD* are reported in infertile and fertilized thalli, which is the opposite to high transcripts for *α-galactosidase* and low for *G3PD* encountered in fertile thalli within visible cystocarps compared to each of their corresponding stages in 35 psu. No significant changes are reported for *MGDGS* and *DGDGS*. It is concluded that cystocarp and thallus development stages affect galactose and glycerides pools with interwoven effects on cell wall polysaccharides.

## 1. Introduction

Red seaweeds synthesize sulfated galactans, such as carrageenan, as a main component of their cell walls, whilst they also store polysaccharides such as floridoside. Sulfated and stored polysaccharides are synthesized from the UDP-hexose pool, which supplies UDP-galactose units to lead the synthesis of the galactan backbone, i.e., sulfated polysaccharides, and to transfer units to glycerol 3-phosphate in order to form floridoside [1]. Sulfated polysaccharides are modified according to the life stages that contribute to the softness of thalli, favoring the localization of reproductive structures. In naturally collected thalli, it was demonstrated that genes encoding proteins for adding and removing sulfated groups to/from the galactan backbone of carrageenan modify their expressions alongside cystocarp development [2]. Moreover, thalli treated exogenously with plant growth volatile regulators, such as ethylene and jasmonates (MEJA), also altered gene expression of biosynthetic precursors of carrageenan synthesis, particularly the synthesis of UDP-hexoses and those in charge of adding and removing sulfated groups to/from the galactan backbone. Additionally, MEJA completely disrupted carrageenan synthesis, as FTIR spectra revealed [3]. The synthesis of carrageenan was also affected by the presence of an exogenous S-source, with implications for the assimilation and activation of the S-source [3,4]. 

Volatile regulators also have an effect on the reproductive stages of red seaweeds, as ethylene increases the number of tetrasporangial branches in the red seaweed *Pterocladiella capillacea* [5,6] and MEJA provokes changes in the cystocarp maturation stages of *Grateloupia imbricata* and favors mixed reproductive stages in *Gelidium arbuscula* [5,7]. Additionally, MEJA induces cystocarp maturation in as little as 48 h (i.e., late mature cystocarps), which is much faster than the 30 days of naturally released spores [7].

**Figure 1:**
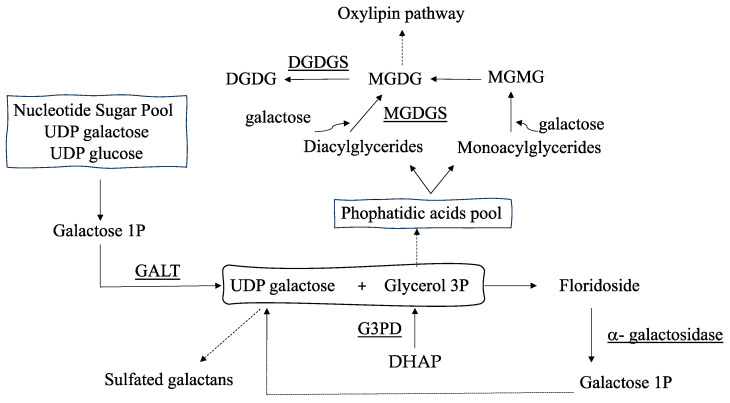
Schematic of biosynthetic pathway for synthesis of floridoside. DHAP, dihydroxyacetone phosphate; GALT, galactose-1-phosphate uridyltransferase; G3PD, glycerol 3-phosphate dehydrogenase; MGDGS, monogalactosyl diacylglyceride synthase; DGDGS, digalactosyl diacylglyceride synthase.

Regarding stored polysaccharides, the most prevalent floridoside is a low-molecular-weight compound that is known to contribute to osmotic acclimation in almost all Rhodophyta [8]. Although the synthesis pathway of floridoside is still unconfirmed [9], it is assumed that its biosynthesis involves the transfer of a galactosyl unit from a pool of UDP- hexoses to glycerol 3-phosphate [10,11](G3P; Figure 1). In reverse, α-galactosidase cleaves galactosyl residues to form UDP-galactose units, which can be used to continue floridoside synthesis and for the synthesis of sulfated galactans [3,4,12] (Figure 1). Accordingly, it was demonstrated that *phosphoglucomutase* (*PGM*) and *galactose-1-phosphate uridyltransferase* (*GALT*), encoding proteins of synthetic precursors and sustaining the pool of UDP-hexoses, altered their gene expression alongside the reproductive stages of thalli of the red seaweed *Grateloupia imbricata* [3,4]. 

On the other hand, glycerol 3-phosphate (G3P) originates from dihydroxyacetone phosphate (DHAP) and is converted to glycerol by means of a phosphatase [13]. Glycerol has been reported as a carbon source for the growth and development of the red seaweed *G. imbricata*. Glycerol provoked a 400% increment in fresh weight and the formation of cell masses after spores were cultured in its presence [14]. Additionally, glycerol has been required for salinity acclimation in other algae [15]. Moreover, G3P connects with the galactoglyceride biosynthetic pathway (Figure 1). In particular, galacto-acylglycerides, of which monogalactosyl diacylglyceride (MGDG) and digalactosyl diacylglyceride (DGDG) are the most abundant, result from the galactosylation of a diglyceride utilizing UDP-galactose [16,17] (Figure 1). Interestingly, MGDG and DGDG participate in the acclimation process, synthesis pathways for plant growth regulators such as jasmonates [17], and changes in the cell membrane fluidity due to alterations in the acylglycerol pool [18].

In these tangled scenarios where sulfated and stored polysaccharides share a precursor (UDP-galactose), and where the synthesis of sulfated polysaccharides is affected by the S-source (reduced vs. oxidized) as well as volatile PGR synthesis through SAM (S-adenosyl methionine), we hypothesized that stored polysaccharides, such as floridoside, may also be affected by the S-source (methionine and SO_4_) and PGRs. Alterations in the floridoside-stored polysaccharide reservoir may be valued through two approaches. The first approach is the synthesis of a pool of hexoses, which forms galactosyl units, and the second is the synthesis of a reservoir of acylglyceride, which supplies the synthesis of glycerol 3-phosphate and favors the flexibility of cell membranes. Accordingly, our aim in this study was to characterize the expression of genes involved in supporting the UDP-galactose pool through synthesis by *GALT* and degradation of floridoside by *α-galactosidase*, and those associated with the acylglyceride reservoir such as *glycerol 3-phosphate dehydrogenase* (*G3PD*), *monogalactosyl diacylglyceride synthase* (*MGDGS*), and *digalactosyl diacylglyceride synthase* (*DGDGS*), during carposporogenesis—induced by ethylene and MEJA—in thalli of the red seaweed *Grateloupia imbricata*, cultured separately in the presence of methionine and SO_4_. 

In addition, gene expression during three reproductive stages of *G. imbricata*, namely, infertile, fertilized, and fertile thalli, was evaluated while increasing the salinity from 35 psu to 65 psu, as *G. imbricata* is an intertidal seaweed. This study sets a benchmark for outlining the connection between the synthesis of sulfated and stored polysaccharides in red seaweeds. Our insights into the genetic mechanisms of the synthesis of valued polysaccharides will contribute to formulating the controlled exploitation of these renewable resources.

## 2. Results

### 2.1. Influence of Ethylene and Meja on Expression of Genes Involved in Floridoside Synthesis alongside Cystocarp Development

**Figure 2:**
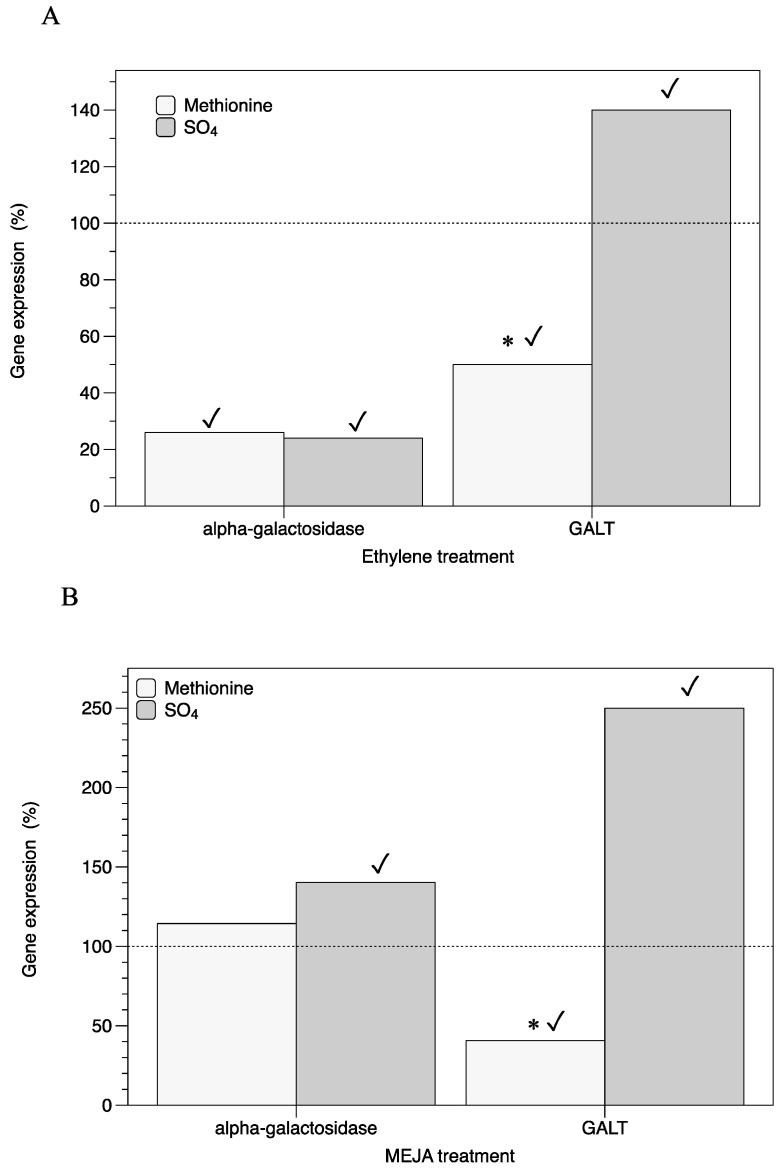
Expression of genes (*alpha-galactosidase* and *GALT*) that encode hexose pool (UDP-galactose) in thalli of *Grateloupia imbricata*. Expression was analyzed in thalli treated with methionine or MgSO_4_ in (**A**) in ethylene-induced fertile thalli at an end time of 10 days (3 days S-source plus 7 days after ethylene treatment), and (**B**) in methylajasmonate (MEJA)-induced fertile thalli at an end time of 5 days (3 days S-source plus 2 days after MEJA treatment). Expressions (copies μL^−1^) are shown as percentages relative to expression in treated thalli at day 3 for methionine and for MgSO_4_, respectively (100%, dashed horizontal line). For methionine, thalli gene expression (i.e., 100%; *p* ≤ 0.01), *GALT* = 120 ± 1.3 ± 1.8 × 10^−4^. For MgSO_4_, *GALT* = 32 ± 1.2 × 10^−5^. Gene expression of pooled samples (i.e., 100%) for *alpha-galactosidase* = 15.4 ± ± 2.1 × 10^−5^ was not affected by S-source. * significant difference (*p* < 0.01) between methionine and MgSO_4_ treatment; ✓ differences between gene and the corresponding control. GALT, galactose-1-phosphate uridyltransferase.

The expression of a gene involved in the synthesis of the pool of UDP-galactose, *galactose-1-phosphate uridyltransferase* (*GALT*), showed a diminution of the transcript number of *GALT* in thalli treated with ethylene (Figure 2A) and MEJA (Figure 2B) in the presence of methionine. Otherwise, the transcript numbers of *α-galactosidase* in thalli treated with ethylene were significantly repressed compared to those of the control (Figure 2A).

**Figure 3:**
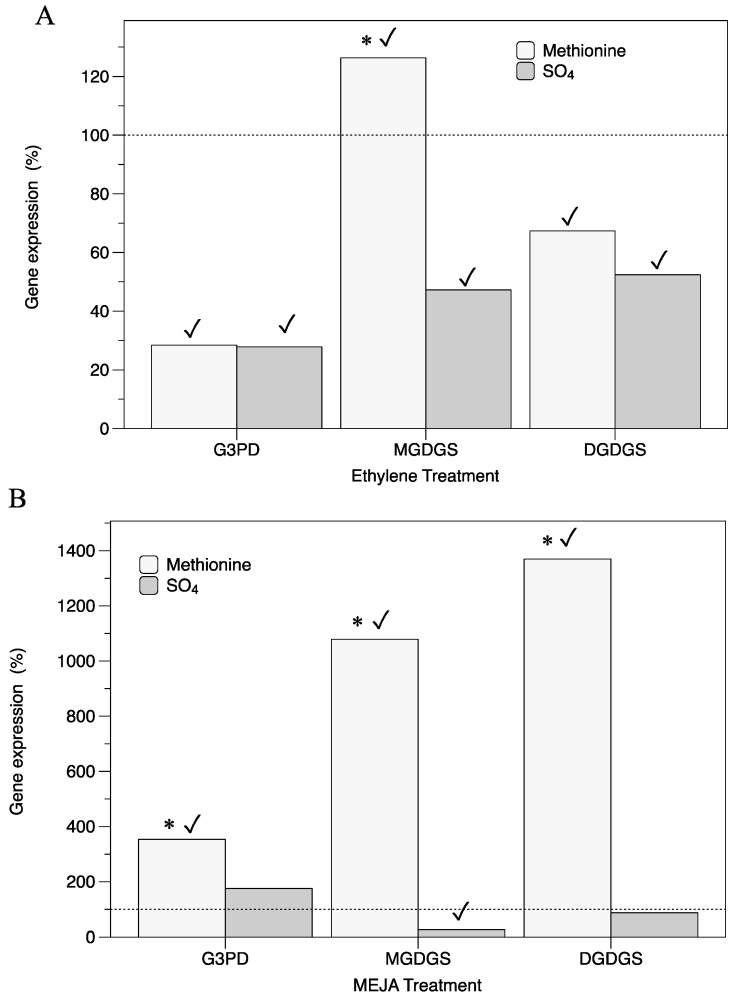
Expression of genes (*G3PD*, *MGDGS*, *DGDGS*) that encode galactosyl–glycerol pool in thalli of *Grateloupia imbricata.* Expression was analyzed in thalli treated with methionine or MgSO_4_ in (**A**) ethylene-induced fertile thalli at an end time of 10 days (3 days S-source plus 7 days after ethylene treatment), and (**B**) in methylajasmonate (MEJA)-induced fertile thalli at an end time of 5 days (3 days S-source plus 2 days after MEJA treatment). Expressions (copies μL^−1^) are shown as percentages relative to expression in treated thalli at day 3 for methionine and for MgSO_4_, respectively (100%, dashed horizontal line). Gene expression of pooled samples (i.e., 100%), *G3PD* = 84.5 ± 1.9 × 10^−5^, *MGDGS* = 91 ± 2.0 × 10^−4^, and *DGDGS* = 93.5 ± 1.87 × 10^−4^. * significant difference (*p* < 0.01) between methionine and MgSO4 treatment; ✓ differences between gene and the corresponding control.

Regarding genes that encode proteins of the galactosyl–glycerol pool such as G3PD, MGDGS, and DGDGS, thalli cultured in ethylene showed significantly repressed expressions for *G3PD* and *DGDGS* in both S-sources (Figure 3). Meanwhile, in the presence of MEJA and methionine, *G3PD*, *MGDGS*, and *DGDGS* were always overexpressed (Figure 3B).

### 2.2. Influence of Salinity on Gene Expression Involved in Floridoside Synthesis

**Figure 4:**
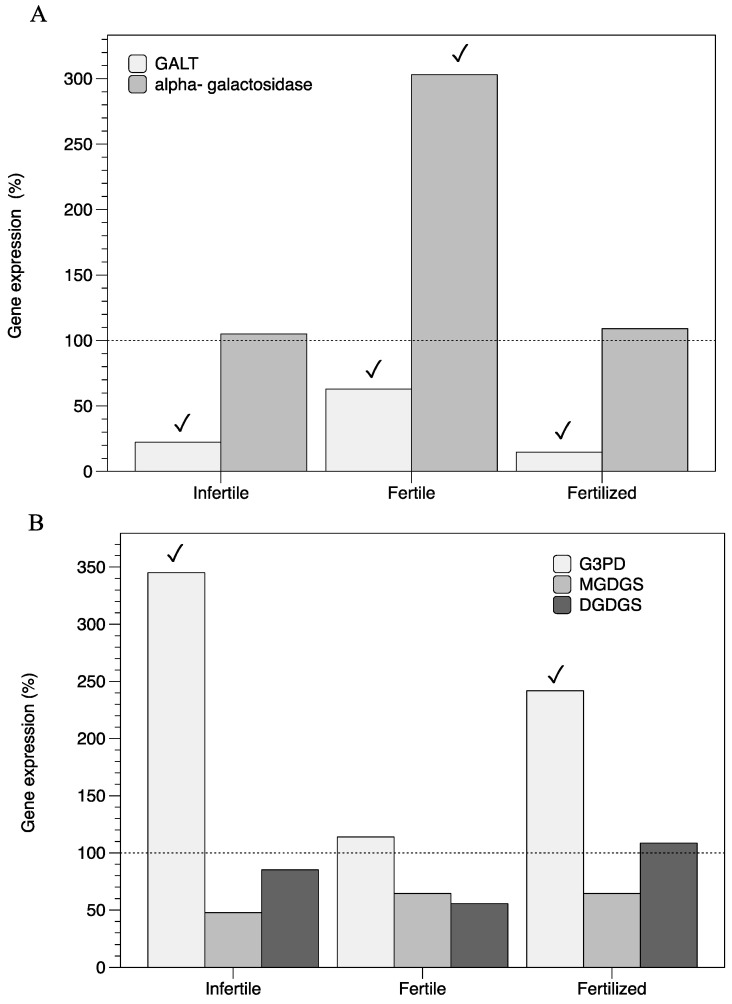
Expression of genes that encode (**A**) hexose pool (*alpha-galactosidase* and *GALT*) and that encode (**B**) galactosyl–glycerol pool (*G3PD*, *MGDGS*, *DGDGS*) under high-salinity conditions (65 psu) in three reproductive stages of thalli of *Grateloupia imbricata* (fertile, fertilized, and infertile thalli). Expressions (copies μL^−1^) are shown as percentages relative to expression in treated thalli at 35 psu (100%, dashed horizontal line). Thalli gene expression (i.e., 100%) *alpha-galactosidase* = 63.22 ± 10^−3^, *GALT* = 296.44 ± 2.50 × 10^−3^, *G3PD* = 48.01 ± 2.7 × 10^−3^, *MGDGS* = 88 ± 3.1 × 10^−4^, and *DGDGS* = 83.33 ± 0.5 × 10^−4^. GALT, galactose-1-phosphate uridyltransferase; G3PD, glycerol 3-phosphate dehydrogenase; MGDGS, monogalactosyl diacylglyceride synthase; DGDGS, digalactosyl diacylglyceride synthase. ✓ differences between gene expression at 65 psu and gene expression at 35 psu.

Increasing the salinity from 35 psu to 65 psu provoked a downregulation of the expression of *GALT* in infertile, fertilized, and fertile thalli (Figure 4A). The gene encoding α-galactosidase was only overexpressed (by nearly three times) in fertile thalli when compared to untreated thalli (35 psu; Figure 4A). Unlike the *G3PD* gene, which was overexpressed in infertile and fertilized thalli, no significant changes were found for the fertile stage, nor were any found for the *MGDGS* and *DGDGS* genes (Figure 4B).

## 3. Discussion

Floridoside, a galactosyl–glycerol-stored polysaccharide of red seaweed, is formed through the conjugation of UDP-galactose and glycerol. Furthermore UDP-galactose, resulting from floridoside’s breakdown by α-galactosidase, contributes to the UDP-hexose pool required for the synthesis of sulfated polysaccharides. Hence, floridoside can act as a dynamic carbon reservoir that can be utilized for polysaccharide biosynthesis. Also, it has been reported that stored polysaccharides play a role in the life cycle [8] and salinity acclimation of seaweeds [19]. Therefore, the formation and degradation of floridoside could be interwoven with sulfated polysaccharides, as carrageenan biosynthesis is also dependent on the hexose pool and reproductive stages of red seaweeds [3,4]. Furthermore, it is relevant that *GALT* expression has been associated with fluctuations in cell wall galactans in the red seaweed *Gracilaria changii* [20] whilst *GALT* expression has also accompanied cystocarp development from induced fertile thalli of *G. imbricata* treated with methionine (Figure 2). Accordingly, the reduced expression of *GALT*, as a gene precursor of galactan synthesis, has been explained by the contribution of methionine to a pool of sulfur organic compounds in algae, which will affect the expression levels of the genes encoding proteins responsible for the conversion of glucose 6P to 1P by PGM and the transformation to UDP-galactose by GALT [3,4]. Thus, *GALT* expression is downregulated in ethylene-induced fertile thalli with cystocarps in the early development stage (Figure 2A), as well as when late cystocarp development stages are reached in the presence of MEJA (Figure 2B). Unlike ethylene, MEJA induced rapid cystocarp maturation and spore release in as little as 48 h in *G. imbricata* thalli [21]. This is in concordance with a previous report, where a *GALT* transcript decrease was correlated with thalli softness, serving to locate reproductive structures, i.e., cystocarps [3,4], as well as constricting the synthesis of UDP-hexoses for galactan backbone synthesis. In the opposite of the case for SO_4_, the reduced expression of *GALT* in methionine could be also explained by a synergistic effect, as methionine is used to synthesize S-methyl methionine (SMM) and S-adenosyl methionine (SAM), whereas SAM is a pivotal compound for ethylene synthesis and a donor of methyl groups for methyl jasmonate synthesis from jasmonic acid [22]. 

Otherwise, a reduction in *α-galactosidase* gene expression in response to a volatile growth regulator may relate to restrained stored polysaccharide mobilization in ethylene-induced fertile thalli (Figure 2A). Early-stage cystocarps in ethylene-induced fertile thalli are encountered 7 days after treatment and continue progressing in maturity until spore release. Hence, thalli softness is still required to locate completely mature cystocarps, which increase in size at that time. Conversely, a significant increase in *α-galactosidase* expression in MEJA (by more than 40% compared to the control) (Figure 2B) would indicate that galactosyl residues could be used to regenerate thalli through cell wall biosynthesis and somehow also prevent thallus injuries as the cystocarps would be completely open and the cell walls would be weakened [7]. Jasmonates are recognized as a disruptor of carrageenan synthesis in *G. imbricata* [4], leading to cystocarp dehiscence through the opening of cystocarps and the release of carpospores in as little as 48 h [7].

The synthesis of glycerol occurs with the formation of glycerol 3-phosphate and consequent dephosphorylation by glycerol 3-phosphate dehydrogenase (G3PD) and by a specific phosphatase [23]. While glycerol is a compound involved in the growth and development of *Grateloupia* spp. [14], glycerol 3-phosphate (G3P) seems to act as a regulator of plant signaling [24].

Upregulation of *G3PD* gene expression in MEJA-induced fertile thalli of *G. imbricata* could explain a supply of G3P for lipid synthesis, as G3PD links carbohydrates and lipid metabolism [25] (Figure 3B). Otherwise, glycerol has been reported as acting like a scavenger to adjust the reducing power in yeast [26], which would also contribute to shielding intermediates from MEJA signaling [21,27]. It is recognized that the synthesis of methyl jasmonate activates the oxidative metabolism of polyunsaturated fatty acids, generating ROSs (in the form of O_2_, H_2_O_2_, or OH^–^) and oxidized derivatives of polyunsaturated fatty acids [28,29]. This would imply that MEJA can provoke changes in the cell membrane fluidity with changes in acylglycerols such as monogalactosyl diacylglycerides (MGDG), and digalactosyl diacylglycerides [18] (DGDG). Indeed, changes in *MGDGS* and *DGDGS* gene expressions indicate the remodeling of cell membranes and allow us to infer that the synthesis of acylglycerols occurs differentially in fertile thalli in the presence of MEJA plus methionine (Figure 3B). The presence of different transcript levels associated with genes encoding lipid remodeling, such as for *MGDGS* and *DGDGS*, has been reported concomitant to differences in tissues [30,31]. This means that in the case of MEJA-induced fertile thalli of *G. imbricata*, high levels of transcripts for *DGDGS* support membrane stability as cystocarps are recognized in a late development stage compared to those in ethylene-induced fertile thalli, where only *MGDGS* is overexpressed (Figure 3A,B). Additionally, these significant gene expression levels in the presence of MEJA and methionine, when compared to those from thalli in the presence of an oxidized S-source, such as SO_4_ (Figure 3B), potentially reveal that ROSs will oxidize excess methionine and form methionine sulfoxide species, which, in turn, can also act on the ROS repository [32]. Likewise, an ethylene double bond will allow this olefin to be easily converted into a range of reactive intermediates [33] and, together with those, methionine provokes fluctuations in the galactosyl–diacyl–glycerol reservoir [18] (Figure 3A).

Together with a likely modulation of the redox state owing to a volatile growth regulator, a scenario of changing osmolarity could also activate *G3PD* in order to constrain an increase in external salinity [34,35,36]. Mechanisms of osmoacclimation have been reported in several *Gracilaria* species as occurring through low-molecular-weight organic solutes, such as galactosyl–glycerols, which are accumulated in hypersaline conditions [37,38,39]. This may justify why *G3PD* was overexpressed in high-salinity-acclimatized thalli of *G. imbricata*, as thallus acclimation transitioned from 35 psu to 65 psu (Figure 4B). Moreover, *Grateloupia imbricata*, an intertidal red seaweed subjected to extreme changes in salinity, irradiance, and temperature, responds to glycerol, which we know as it is successfully used for heterotrophic growth of *G. imbricata* (formerly *G. doryphora*) [14]. Additionally, the expression of the *α-galactosidase* gene, encoding the floridoside-degrading protein, is constant in a high-salinity situation in infertile and fertilized thalli (Figure 4A). In contrast, in fertile thalli, high transcripts of *α-galactosidase* (Figure 4A) and unaltered *G3PD* (Figure 4B) could point to the cleavage of galactosyl residues from the floridoside reservoir to refurbish the cell wall of thalli with mature cystocarps. In higher plants, galactosidase activity is associated with a reduction in the level of galactosyl residues occurring in ripening seeds and different profiles have been reported of gene expression for *α-galactosidase* in several tissues during the maturation and germination of seeds [40]. In red seaweeds, differential behavior for stored polysaccharides seems to occur according to the development stages of thalli, namely, infertile, fertile, and fertilized thalli. Thus, floridoside is accumulated in infertile and fertilized (i.e., non-visible cystocarps; high expression for *G3PD* and low for *α-galactosidase*; Figure 4A,B) and degraded in fertile thalli (i.e., visible and late-mature cystocarps; low expression for *G3PD* and high for *α-galactosidase*; Figure 4A,B), all in order to render galactosyl units available for the synthesis and restoration of the cell wall during the reproduction process of red seaweeds. 

In conclusion, the synthesis and degradation of floridoside are interwoven with sulfated polysaccharides, serving to refurbish thalli according to the development stages of the red seaweed. This implies balanced gene expression through the downregulation of hexose pool transcripts (i.e., no units for the galactan backbone) to soften the cell wall and upregulation of those in charge of sustaining the galactosyl–glycerol pool to support fluidity and stabilization of cell membranes during late events of the carposporogenesis of *G. imbricata* induced by volatile growth regulators. Moreover, floridoside accumulation seems to be controlled for the reproductive stages of *G. imbricata* rather than salinity changes, as mobilization of the hexose pool is assumed during changes in *α-galactosidase* gene expression alongside the three development stages of thalli, which transition as infertile, fertilized, and fertile thalli.

Understanding the gene mechanisms responsible for driving floridoside accumulation and degradation and for cell membrane flexibility during the reproductive events of red seaweeds, presents a unique opportunity to further our control of the biosynthesis of carrageenan, which is used as a marine source in diverse industries.

## 4. Materials and Methods

### 4.1. Plant Material and Culture Conditions

Infertile, fertile, and fertilized thalli from the carragenophytic *G. imbricata* were collected along the northeast coast of Gran Canaria in the Canary Islands. 

**Figure 5:**
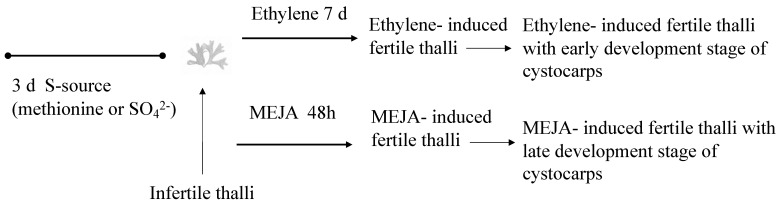
Scheme showing timeline for determination of gene expression (thin arrow) under ethylene and methyl jasmonate (MEJA) action in *Grateloupia imbricata*. This is infertile thalli treated with methionine or MgSO_4_ for 3 days; ethylene-induced fertile thalli at an end time of 10 days (3 days S-source plus 7 days after ethylene treatment); and in MEJA-induced fertile thalli at an end time of 5 days (3 days S-source plus 2 days after MEJA treatment). Controls are thalli treated for 3 days with the corresponding S-source.

Concerning the evaluation of the role of a plant growth volatile regulator on floridoside synthesis and considering the role of the S-source in polysaccharide synthesis [3,4,41], infertile thalli were placed in 500 mL vessels (3 g per vessel) and cultivated separately, either with a reduced source of S such as methionine (10 mM) or oxidized as magnesium sulfate (1.6 mM) for 3 days. Then, infertile thalli were sprayed three times with a solution of 100 μM MEJA (Sigma Co., St Louis, MO, USA) in 0.01% (*v*/*v*) ethanol in an autoclaved seawater solution [21] to elicit cystocarps. The thalli were kept for 1 h under a light source of 50 μmol photons m^−2^ s^−1^ and continued to be cultivated for 48 h (henceforth, MEJA-induced fertile thalli with a late development stage of cystocarps; Figure 5). 

Also, we triggered infertile thalli to elicit cystocarps by applying ethylene [42] (99.9% purity, Carburos Metálicos SA, Barcelona, Spain) to the 500 mL sealed vessels for 15 min at a flow rate of 0.5 L min^−1^. The thalli continued to be cultivated for 7 days (henceforth, ethylene-induced fertile thalli with an early development stage of cystocarps; Figure 5). 

Thallus development from infertile to induced fertile thalli was verified through the gene expression of *ornithine decarboxylase* (*ODC*), which acts as a reproduction marker gene in red seaweeds [7,21,42]. The *ODC* gene expression was always downregulated in fertile thalli (i.e., 2.45 ± 0.15 × 10^−2^ copies μL^−1^ in infertile thalli and 0.99 ± 0.05 × 10^−2^ copies μL^−1^ in fertile thalli) [3].

**Figure 6:**
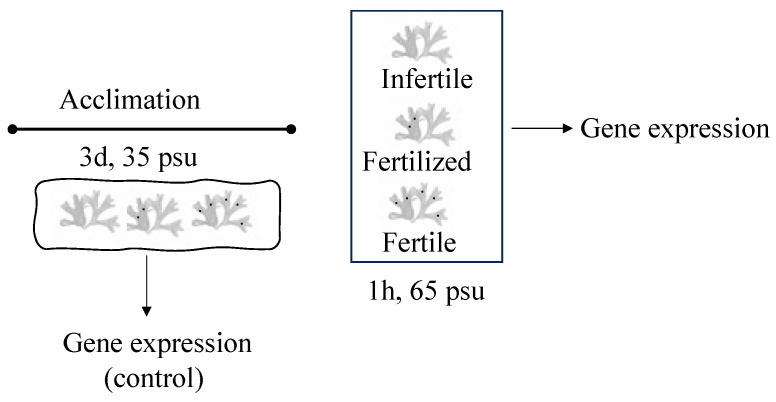
Scheme showing timeline for determination of gene expression (thin arrow) under high-salinity conditions in *Grateloupia imbricata*. Thalli were acclimatized at 35 psu for 3 days (salinity control) and then sorted according to reproductive stages (fertile, fertilized, and infertile thalli) and cultivated at 65 psu for 1 h.

In order to assay the effect of salinity on the synthesis of floridoside, thalli were acclimated at 35 psu for 3 days to avoid bias as *G. imbricata* is an intertidal seaweed. Then, thalli were placed separately into three aquaria at 65 psu for 1 h, allowing us to classify them into fertile thalli, which showed axes with cystocarps; fertilized thalli, which displayed axes without visible cystocarps from the same individual with axes with cystocarps; and infertile thalli, which showed no cystocarps (Figure 6). All thalli were maintained at 20 ± 2 °C under an 18 h light (50 μmol photons m^−2^s^−1^): 6 h dark photoperiod in a growth chamber.

### 4.2. Influence of Ethylene and Methyl Jasmonate in Floridoside Synthesis 

To value storage polysaccharide synthesis (i.e., floridoside synthesis), two approaches were deemed taking into consideration that floridoside synthesis is managed by 1) a pool of UDP-galactose and UDP-glucose and 2) a galactosyl–glycerol pool (Figure 1). Thus, the expression levels of *GALT*, which is in charge of forming UDP-galactose from galactose-1-P, and *α-galactosidase*, which degrades floridoside, were measured. Also, genes encoding glycerol 3-phosphate dehydrogenase (G3PD, in charge of supplying glycerol 3-P) and those providing monogalactosyl diglyceride through *MGDG synthase (MGDGS)* and digalactosyl diglyceride by *DGDG synthase (DGDGS*) were analyzed as somehow they are involved in the synthesis of the galactosyl–glycerol pool (Figure 1).

All gene expression levels were valued in fertile thalli induced by MEJA and ethylene (Figure 5). Control samples (untreated and treated with each one of the S-sources) were processed in parallel. All samples were assayed in triplicate with two independent replicates for each experiment. At the end of the investigation period, samples of the thalli were frozen at −80 °C until the isolation of RNA.

### 4.3. Influence of Salinity Changes in Floridoside Synthesis

Transcripts of all genes detailed above were also measured in infertile, fertilized, and fertile thalli after acclimation for 3 days at 35 psu (control) and at 65 psu, to assess the salinity effect on floridoside synthesis (Figure 6). Samples and the control were assayed in triplicate with two independent replicates for each experiment. At the end of the investigation period, samples of the thalli were frozen at −80 °C until the isolation of RNA.

### 4.4. RNA Extraction

The total RNA was separately extracted from the upper-half regions (100 mg) of thalli using 1 mL of Tri-Reagent (Sigma, St. Louis, MO, USA), according to the manufacturer’s instructions. The isolated RNA samples were individually suspended in 20 μL of 1 M Tris-HCl (pH 8) and 0.5 M EDTA, and they were treated with DNase (1 Umg^−1^, Promega, Madison, WI, USA) to destroy contaminating DNA. The total RNA was quantified using a TrayCell cuvette and Beckman Coulter DU 530 spectrophotometer. Next, RNA extracted from each sample (~1 μg) was reverse transcribed in the presence of oligo (dT) and primers with randomly generated sequences from an iScript cDNA synthesis kit (Bio-Rad, Hercules, CA, USA). The reverse transcription procedure was carried out at 25 °C for 5 min, 42 °C for 30 min, and 85 °C for 5 min. The integrity of the cDNA was validated using a NanoDrop spectrophotometer (Thermo Fisher Scientific, Waltham, MA, USA). The products were kept at 4 °C until used.

### 4.5. Droplet Digital PCR (ddPCR) Primers and Protocol Implementation

For quantification of each target transcript by ddPCR, QX200 ddPCR EvaGreen Supermix (Bio-Rad) was used according to the manufacturer’s instructions. Briefly, for each sample, a PCR reaction mix (final volume 20 μL) was prepared containing 1.5 μL of cDNA, 10 μL of QX200 ddPCR Eva Green Supermix, and 0.22 μL of each primer (10 μM), and then was loaded into a cartridge. Then, an oil droplet (70 μL) was loaded into each cartridge, and the cartridge was covered with a gasket. Each cartridge was individually introduced into the droplet generator, and finally, droplets of ~40 μL were transferred to the amplification plate. For each gene, three replicates were analyzed for each sample treated with MEJA and ethylene, acclimated to salinity, and we analyzed control samples accordingly. Primers for ddPCR were designed from cDNA sequences of the *G. imbricata* transcriptome (Table 1). PCR amplification was performed with a C1000 Touch Thermal Cycler (Bio-Rad) using the following conditions: an initial step at 95 °C for 5 min; followed by 40 cycles at 95 °C for 30 s, an experimentally determined annealing temperature for each gene for 1 min, and 72 °C for 45 s; then, a single step at 4 °C for 5 min; and a temperature ramping from 4 °C to 90 °C at a rate of 2 °C s^−1^ for 5 min. After amplification, each sample was quantified using QuantaSoft v1.7.4 software (Bio-Rad). Data from merged wells (corresponding to each group of replicates) were retrieved, and the concentration of each group was given as the average number of transcript copies per μL.

### 4.6. Data Analysis

Gene expression (transcript copies × μL^−1^) is reported as the mean ± standard deviation (SD). Statistical comparisons of concentrations were performed using R software (https://www.r-project.org; accessed on 6 July 2023). A one-way ANOVA followed by the post hoc tests Tukey HSD and Dunnett T3 was used to detect significant differences (*p* ≤ 0.01) between genes and their respective controls for each S-source (methionine and SO_4_). 

## Figures and Tables

**Table 1:** Sequences of the forward (F) and reverse (R) primers for each gene involved in synthesis of hexose pool (i.e., *alpha-galactosidase* and *galactose-1-phosphate uridylyl transferase* (*GALT*), and in synthesis of galactosyl–glycerol pool, namely, *glycerol 3-phosphate dehydrogenase* (*G3PD*), *monogalactosyl diacylglycerol synthase* (*MGDGS*), and *digalactosyl diacylglycerol synthase* (*DGDGS*)).

Gene	Primer Name	Sequence (5′-3′)
Synthesis of hexose pool		
*alpha-galactosidase*	AG-2468F AG-2468R	CTGTCAAGTTCCCGGATTCTC TTCCTGCTGAAAGTCCCATTAG
*galactose-1-phosphate uridylyl transferase* (*GALT*)	G1PU-1681F G1PU-1681R	GTAGTAGATGCCTGGTGTGATG CATATCTGGCCATGAGGATGAG
Synthesis of galactosyl–glycerol pool		
*glycerol 3-phosphate dehydrogenase* (*G3PD)*	G3PD-7275F G3PD-7275R	ACCTATCGGGTCCTTCATTTG GGATGAGAACATGTCACCTAGAG
*monogalactosyl diacylglyceride synthase* (*MGDGS*)	MGS-2948F MGS-2948R	TCCCGTTTAATCACTTCCCTTC ACTAAACGCGGTCTCAGTAATC
*digalactosyl diacylglyceride synthase (DGDGS*)	DGS-3637F DGS-3637R	GTCCAATCCCAATCGAAGAGAG CTCAGCCAGACAATTCCGATAA

## Data Availability

The original data presented in the study are included in the article; further inquiries can be directed to the corresponding author.
